# Noninvasive low-cycle fatigue characterization at high depth with photoacoustic eigen-spectrum analysis

**DOI:** 10.1038/s41598-018-26140-x

**Published:** 2018-05-17

**Authors:** Xiaoxiang Gao, Chao Tao, Rong Zhu, Xiaojun Liu

**Affiliations:** 1 MOE Key Laboratory of Modern Acoustics, Department of Physics, Collaborative Innovation Center of Advanced Microstructures, Nanjing University, Nanjing, 210093 China; 2 Department of Materials Science and Engineering, Nanjing University of Science and Technology, Nanjing, 210094 China

## Abstract

In this work, photoacoustic eigen-spectrum analysis was proposed for noninvasively characterizing the mechanical properties of materials. We theoretically predicted the relationship between the photoacoustic eigen-spectra of cylindrical optical absorbers and their mechanical properties. Experimental measurements of eigen-spectra extracted from photoacoustic coda waves agreed well with the theoretical predictions. We then applied the photoacoustic eigen-spectrum analysis for contactless monitoring of low-cycle fatigue damage. Experiments showed that the photoacoustic eigen-spectra were closely related to the degree of low-cycle fatigue. This study might enhance the contrast of photoacoustic imaging ford mechanical characterization.

## Introduction

Photoacoustic imaging (PAI), based on the photoacoustic effect, is a hybrid technique that combines the advantages of high ultrasonic resolution in deep tissues and strong optical contrast^1–24^. When a material is irradiated by pulsed lasers, optical energy is converted to heat, and ultrasound waves are sequentially induced after thermoelastic expansion. Benefiting from multiple physical effects during the generation and propagation of photoacoustic signals, PAI can extract various chemical and physical parameters of materials, such as chemical components^1–3^, velocity^4^, elasticity^5–8^, microstructure^9–11^ and temperature^12^, thereby providing various imaging contrasts. These rich contrasts not only show great potential for the non-invasive characterization of materials^13–15^ but also have valuable biomedical applications, including breast imaging^16^, vasculature visualization^17,18^, osteoarthritis assessment^19,20^, small-animal whole-body imaging^21,22^, dental implantation^23^, and drug delivery monitoring^24^.

Mechanical characteristics are fundamental attributes of materials. They are crucial to monitoring structural strength, fatigue and fracture. Additionally, the mechanical properties of tissues are important biological indicators of many diseases, such as atherosclerosis^25^, osteoporosis^26^, and bone fracture^27^. Consequently, these properties play an important role in the fields of materials science^28^, engineering^29^, biomedicine^25–27^, etc. Many studies have involved the monitoring of the mechanical characteristics and fatigue of various materials^30–45^. Some methods generate mechanical resonance in specimens by a bimorph piezoelectric plate-like actuator^31^ or a hammer^32,33^. Displacement is then achieved by contact measuring using sensors attached to specimens^32,33^. Sound waves radiated from an ultrasonic transducer can also stimulate resonance when the frequency of incident waves equals the resonance frequency of a specimen^34^. However, the strain of a specimen is transferred by a rod and detected by a displacement transducer. These methods rely on contacting vibration excitation or contacting signal detection. All optical resonant ultrasound spectroscopy (RUS)^35–37^ and laser ultrasonic methods^38–41^ provide contactless techniques for the measurement of mechanical properties. These methods require direct detection of the surface vibration of samples via optical methods. Electromagnetic acoustic resonance (EMAR) provides a contactless technique for fatigue evaluation^42–45^. In this method, an electromagnetic acoustic transducer based on the magnetostrictive effect is employed to excite axial–shear acoustic resonance and receive resonant frequencies. Benefitting from the use of surface-wave resonance and an electromagnetic acoustic transducer, EMAR is a non-contact method and highly sensitive in evaluating fatigue damage. The technique can monitor fatigue online without interrupting fatigue tests, and it has the potential to characterize inhomogeneous microstructures in the radial direction. However, this method is usually suitable only for ferromagnetic metals. Therefore, a new technique for monitoring the mechanics and fatigue of materials is required.

Photoacoustic methods have also demonstrated their capacity to evaluate the mechanical properties of materials. According to the quantitative relationship between the viscoelasticity and the phase delay of the strain response to the stress of tissues, the viscoelasticity can be estimated from photoacoustic signals detected from tissues^7,8^. Based on this method, photoacoustic viscoelasticity imaging in atherosclerosis^7^ and intraluminal tissues^8^ has been well studied. Recently, we observed a physical phenomenon, named the photoacoustic eigen-spectrum, in the photoacoustic coda waves of spherical optical absorbers^6^. It has been demonstrated that the eigen-vibration information and mechanical properties of a spherical object are imprinted in the generated photoacoustic coda waves. These previous studies have demonstrated the proficiency of photoacoustic methods in mechanical characterization.

In this work, we studied the photoacoustic eigen-spectra of cylindrical optical absorbers and characterized the fatigue of materials by using photoacoustic eigen-spectrum analysis. Materials fatigue refers to the weakening of properties caused by cyclic loading^29^. This phenomenon has attracted much attention because at least 50 percent of mechanical failures result from fatigue^46^. In medicine, monitoring and assessing bone fatigue^47^ or intravascular stents^48^ are closely connected to evaluating human health. First, we theoretically predicted the photoacoustic eigen-spectra from cylindrical optical absorbers in deep tissue-like media. Then, photoacoustic waves of different wires were detected in experiments, and eigen-spectra were extracted from the photoacoustic coda waves to verify the theoretical predictions. Finally, we applied photoacoustic eigen-spectrum analysis to monitor the low-cycle fatigue of wires.

## Results

### Eigen-spectra prediction of cylindrical optical absorbers

**Figure 1:**
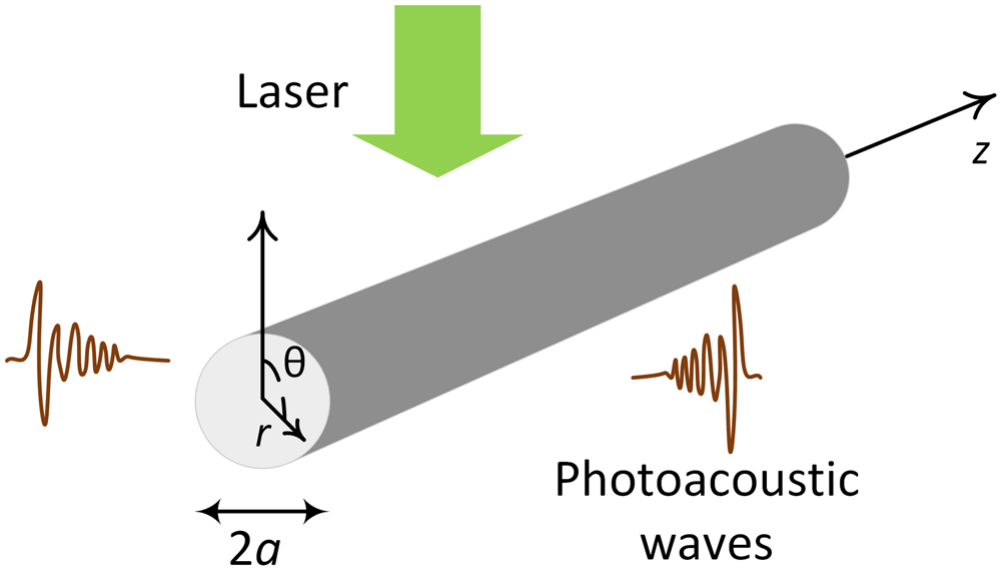
Schematic diagram of photoacoustic wave generation.

A schematic diagram of the photoacoustic generation from an infinite, isotropic and homogeneous elastic cylinder with radius *a* is shown in Fig. 1. After laser pulse illumination, the cylinder absorbs the incoming optical energy, experiences thermoelastic expansion, and finally emits photoacoustic waves to the surrounding media. The laser generates a wideband photoacoustic head wave. After the laser pulse vanishes, the absorber continues to vibrate and emits a photoacoustic coda wave following the head wave, which contains narrow-band spectral lines determined by the eigen-modes of the absorber^6^. Therefore, investigating the photoacoustic eigen-spectrum requires prediction of the eigen-frequencies. The wave equation in terms of the displacement **u** is

1 $$(\lambda +2\mu )\nabla (\nabla \cdot {\bf{u}})-\mu \nabla \times \nabla \times {\bf{u}}={\rho }_{c}\frac{{\partial }^{2}{\bf{u}}}{\partial {t}^{2}}$$

where *λ*, *μ* are Lamé constants and *ρ*_*c*_ is the density of the cylinder. The speeds of longitudinal and shear waves can be expressed as *c*_*d*_ = [(*λ* + 2*μ*)/*ρ*_*c*_]^1/2^ and *c*_*s*_ = (*μ*/*ρ*_*c*_)^1/2^, respectively. By introducing potential functions and applying accurate boundary conditions, the eigen-frequencies should obey the equation^49^

2 $$\det ({\bf{D}})=0.$$

The elements of **D** are listed in the Methods section. As indicated by **D**, the eigen-frequencies are determined by the mechanical properties of the cylinder. Therefore, quantification of the eigen-frequencies could be a method for mechanical characterization. Newton’s iteration method is used to calculate eigen-frequencies of three types of cylindrical optical absorbers made of brass, steel and tungsten. The parameters required for computation are presented in Table 1. In this work, eigen-modes are named according to the convention established in ref.^35^. Because we assume the cylinder to be infinite, the calculated eigen-modes are cross-section modes^35^. The modes are Rayleigh (R) modes or wave gallery (WG) modes, which are denoted R(*m*,*n*) modes and WG(*m*,*n*) modes, respectively. The integer *m* represents the mode order, and *n* is the frequency number of the corresponding *m*. When the tangential displacement *u*_*θ*_ equals 0, the modes are called longitudinal (L) modes. The first longitudinal mode is expressed as L(0,1). Calculation results are not listed here but displayed later to compare with the experimental results.

**Table 1:** Materials parameters of different wires.

Parameters	Brass	Steel	Tungsten
*a* (mm)	0.25	0.3	0.25
*ρ*_*c*_ (10^3^ kg/m^3^)	8.5	7.8	19.25
*c*_*d*_ (10^3^ m/s)	4.37	5.85	5.18
*c*_*s*_ (10^3^ m/s)	2.10	3.24	2.87

### Photoacoustic measurement system

**Figure 2:**
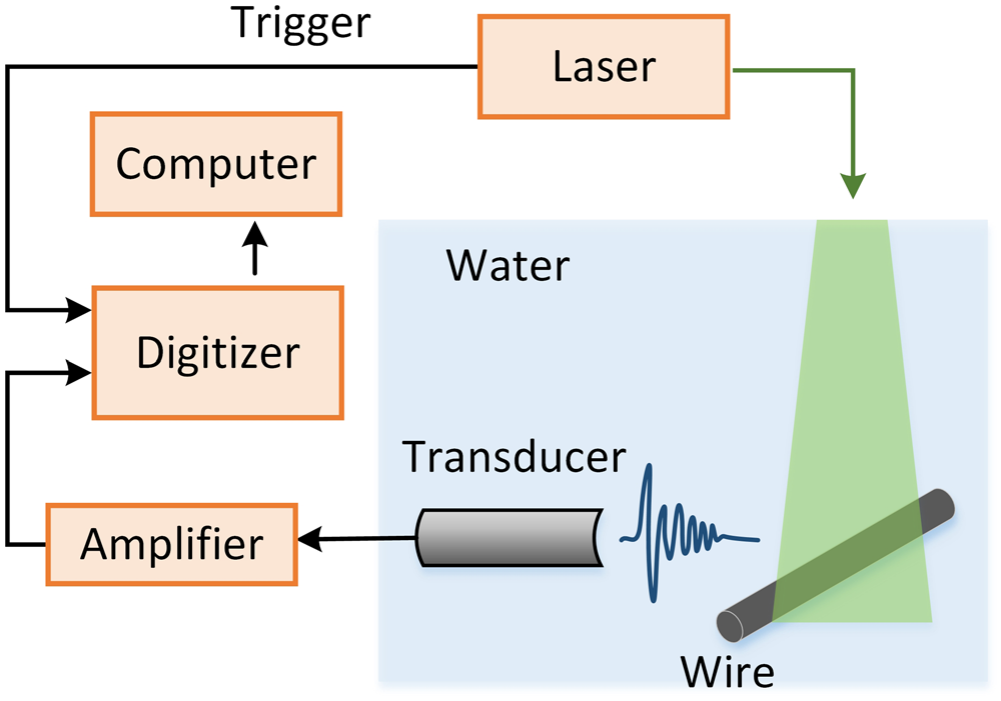
Schematic of the photoacoustic measurement system.

We detected the photoacoustic signals of different wire samples with a photoacoustic measurement system. The experimental setup is illustrated in Fig. 2. Samples were illuminated by a Q-switched Nd:YAG laser with a wavelength of 532 nm and a pulse width of approximately 8~10 ns. A laser spot with diameter of approximately 1 cm was illuminated on the surface of each wire. An ultrasound transducer (V310, Panametrics, 4.39 MHz center frequency, 100.1% relative bandwidth at −6 dB) was used to receive ultrasound signals. The signals were amplified (SA-230F5, NF) and recorded (PCI-5105, NI) at a sampling frequency of 60 MHz. We separately immersed three different types of wires in water to perform the experiments; the wires were made of brass, steel, and tungsten. Each wire had a length of approximately 10 cm, guaranteeing that the ratio of length (under laser illumination) to diameter was greater than 16. Under these conditions, the wires could be approximated as cylinders of infinite length. Therefore, cross-section eigen-modes were mainly generated in this study. The radii and mechanical parameters of the samples, including density, longitudinal wave speed and shear wave speed, are listed in Table 1. The experimental measurements were repeated at 10 different positions on each wire, and each signal was calculated as the average of 50 experiments to reduce the noise in each measurement. During the experiments, the ultrasound transducer used for signal detection was approximately two centimeters from the specimen. This distance could reduce the signal amplitude due to acoustic absorption but did not shift the frequency values.

### Photoacoustic eigen-spectrum extraction

**Figure 3:**
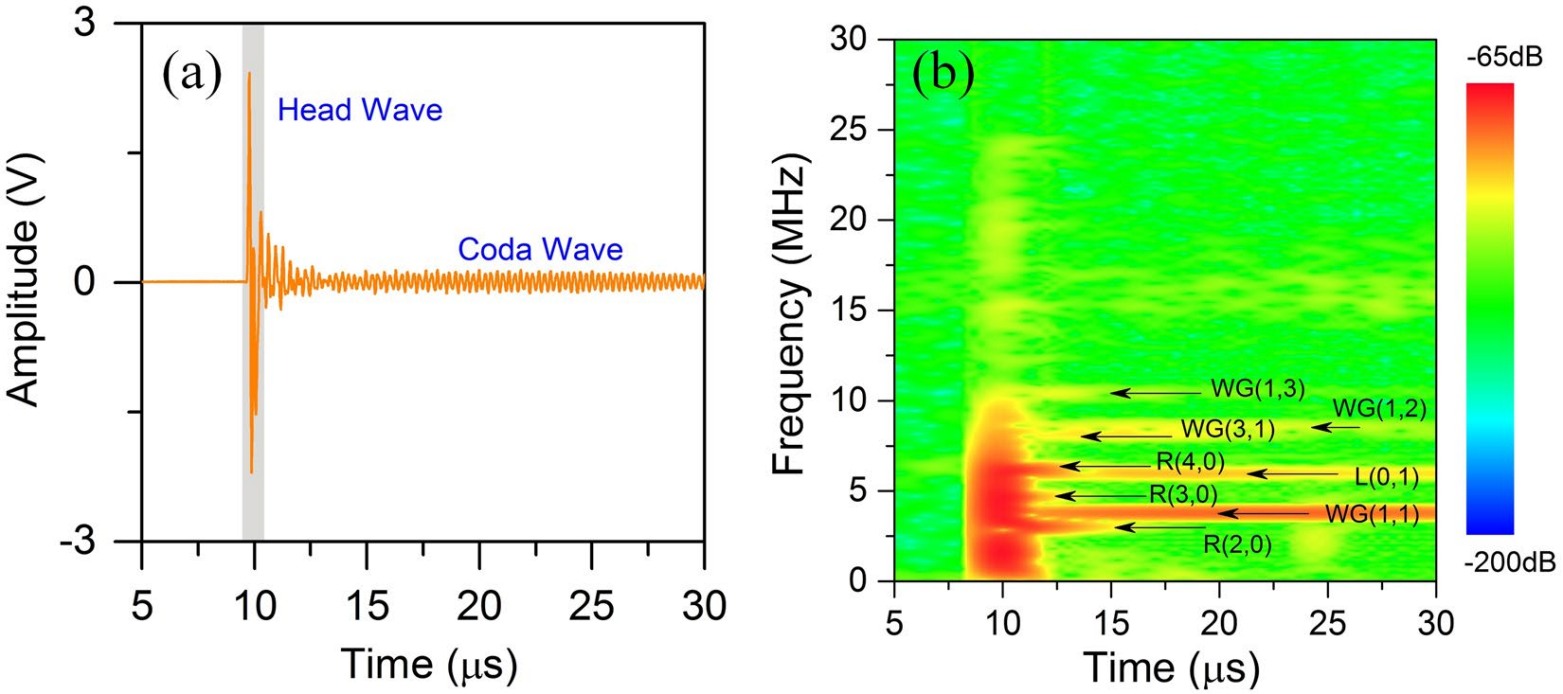
The extraction of the photoacoustic eigen-spectrum from experimental photoacoustic waves. (**a**) A typical temporal wave of the brass cylinder. The head wave has a higher amplitude, which is followed by the coda wave. (**b**) The time-frequency map of the signal in (**a**). The spectral lines represent different eigen-modes.

**Figure 4:**
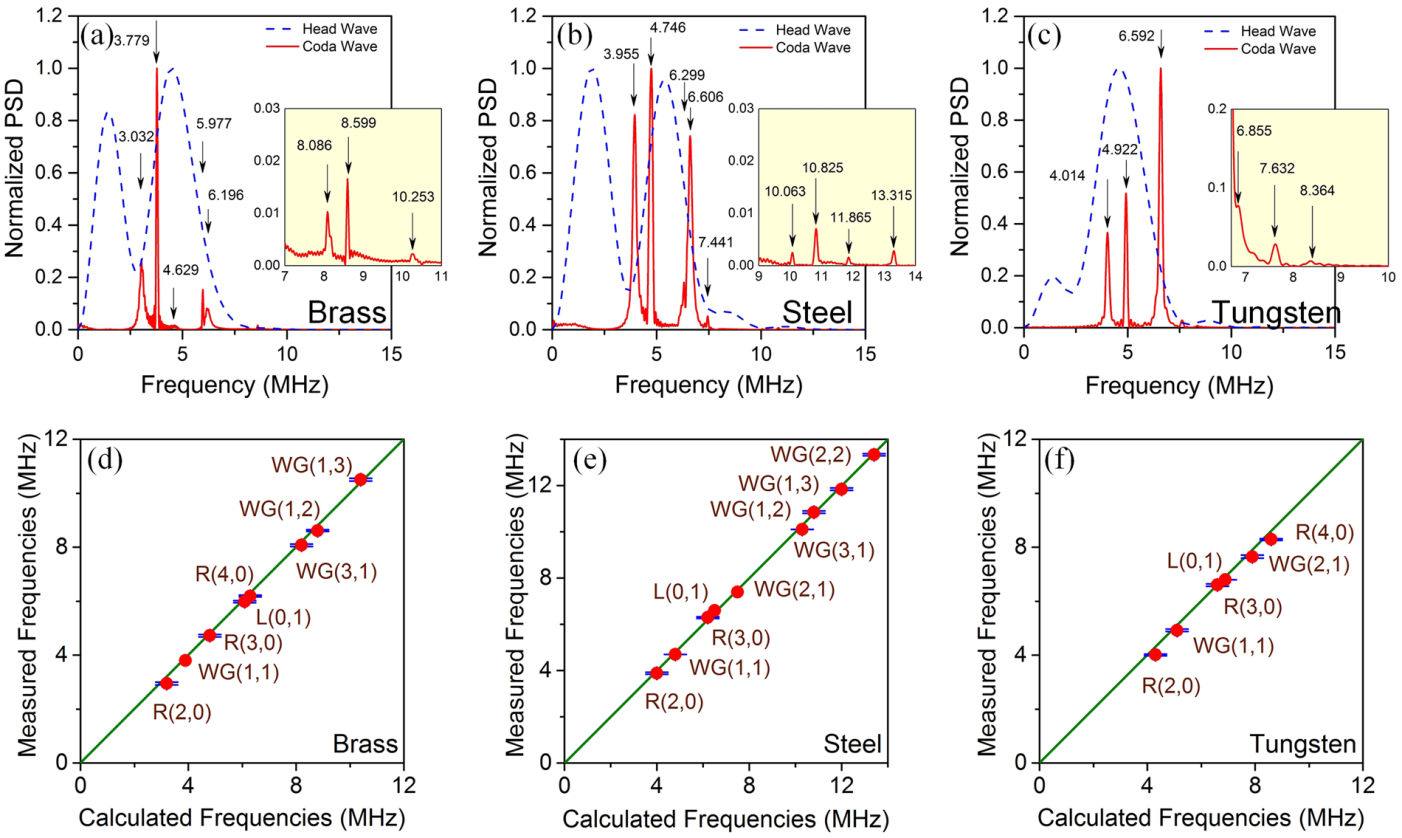
Extraction of eigen-frequencies of different materials from experimental photoacoustic signals. (**a**–**c**) Comparisons of the normalized PSDs between the head waves and coda waves of brass, steel, and tungsten wires, respectively. (**d**–**f**) Mean values of measured eigen-frequencies against the calculations for brass, steel, and tungsten wires, respectively. Error bars denote the standard variations.

Figure 3 shows the photoacoustic eigen-spectrum extracted from the measured signals. One typical temporal waveform of the brass wire is drawn in Fig. 3(a). The waveform has a signal-to-noise ratio of approximately 30 dB. The beginning stage of the wave has a higher amplitude than the rest of the wave, which was directly excited by the pulsed laser. This broadband wave, referred to as the head wave, is related to the dimensions and the optical absorption coefficient of the cylinder. Such waves have demonstrated great capacity for microstructure characterization of deep tissues at low frequency^10,11^. Beyond the head wave lies a long-duration wave with attenuated amplitude. This feature indicates that, when the laser pulse vanishes, the cylinder continues vibrating and emitting sound waves. The coda wave should have the same frequencies as the vibration of the cylinder, which are expected to be the eigen-frequencies. The time-frequency map of the corresponding wave is calculated and presented in Fig. 3(b). It is clear that there are several distinct spectral lines in the coda wave, which are demonstrated to well coincide with the theoretically calculated eigen-frequencies. Figure 4(a–c) present the normalized power spectrum density (PSD) of the head waves and coda waves of all three samples. The results confirm that the head waves are wideband photoacoustic waves, whereas the coda waves with narrow spectral lines correspond to periodic waves of eigen-vibrations. Figure 4(d–f) compare the eigen-frequencies between the experimental values and theoretical predictions for all three wires. Each scatter point denotes one eigen-mode. The error bars show the standard variations of the measured eigen-frequencies. The good agreement between the two sets of results confirms the validity of the theoretical analysis.

### Preparation of low-cycle fatigue damaged samples

**Figure 5:**
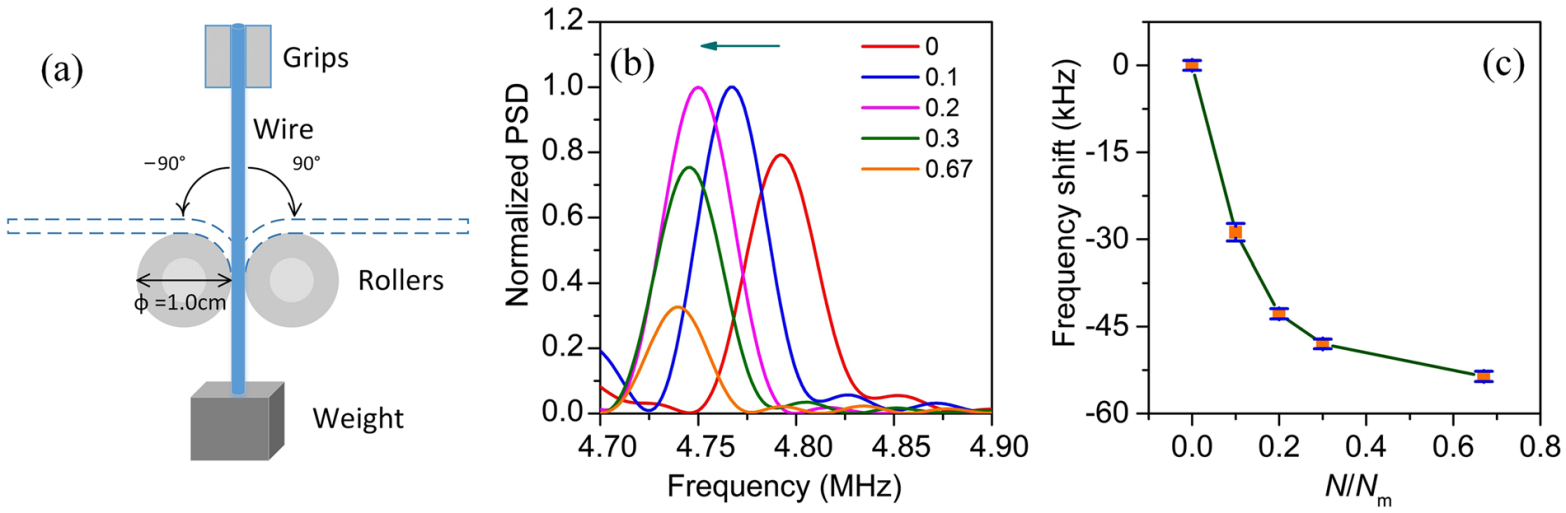
Eigen-frequency shifts of low-cycle fatigue damaged steel wires. (**a**) Diagram of the bending fatigue machine for the wire. (**b**) The normalized PSDs of coda waves of damage-free and low-cycle fatigued steel wires. The frequency band is limited to the range between 4.70 and 4.90 MHz, where the mode WG(1,1) lies. (**c**) The eigen-frequency shift values of WG(1,1) versus different bending fractions, where *N*_*m*_ = 30.

We applied photoacoustic eigen-spectrum analysis to monitor the low-cycle fatigue of a wire made of AISI 1070 high-carbon steel. A bending fatigue scheme was adopted to induce fatigue in the wire^50^, as shown in Fig. 5(a). One end of the wire was held by grips attached to a bending arm. Two rollers (1 cm in diameter) restrained the middle part of the wire with the contact boundaries free. The lower end was bundled with a suspended weight (1 kg) to keep the lower part vertical and to apply steady tension within the wire. In one bending cycle, the wire was bent 90° and −90° and returned to the initial position. We bent one steel wire with a diameter of 0.6 mm over 3, 6, 9, and 20 cycles until the wire approached fracture. As the wire broke after 30 cycles of bending, the maximum number of cycles *N*_*m*_ was 30. In our fatigue test, the specimen was plastically deformed in each loading cycle and underwent a small number of cycles to failure. This type of fatigue is referred to as low-cycle fatigue. The wire was measured five times by repeating the previously described photoacoustic measurement procedures after performing different numbers of bending cycles. The length of the bent part was approximately 7.85 mm, nearly 13 times the diameter of the wire; thus, the bent part could still be considered a long, homogeneous cylinder. During the measurement of photoacoustic signals, the ultrasound transducer was directed toward the bent part. Therefore, the acoustic waves emanated only from the bent part.

### Low-cycle fatigue characterization by photoacoustic eigen-spectrum analysis

The normalized PSDs of the photoacoustic coda waves of the raw and bent wires are drawn in Fig. 5(b). In particular, the frequency range from 4.70 to 4.90 MHz is shown, over which the mode WG(1,1) lies. The WG(1,1) mode was chosen for two reasons. First, WG(1,1) showed the highest amplitude among all modes. The other modes were weak or were even missed by the fatigue test. Second, WG(1,1) remained table for the longest period, as shown in Fig. 3(b). Such a long-lived mode allows for the calculation of its eigen-frequency within the widest window. A wide time window means high frequency precision. Therefore, the analysis of mode WG(1,1) can help improve the accuracy of eigen-frequency extraction and the sensitivity of fatigue characterization. We introduced the ratio of the actual number of cycles to the maximum number of cycles *N*/*N*_*m*_ to quantify the degree of low-cycle fatigue. In Fig. 5(b), the values 0, 0.1, 0.2, 0.3 and 0.67 in the legend refer to this ratio. The curve at a value of 0 denotes the unbent wire, while the other curves correspond to the wires submitted to different degrees of fatigue. It is shown that all the peaks of WG(1,1) move to lower frequencies. Figure 5(c) quantifies the frequency shift versus the bending ratio. The ordinate value is calculated as the frequency minus the mean value of the eigen-frequencies of the wire with no bending. Clearly, as the number of bending increases, fatigue accumulates and the frequency shift increases. The mean eigen-frequency shifts for the bent wires are approximately −29 kHz, −43 kHz, −48 kHz and −54 kHz. The error bars denote the standard deviations. The eigen-frequency dropped significantly at the beginning and tended to become invariant in later stages.

**Figure 6:**
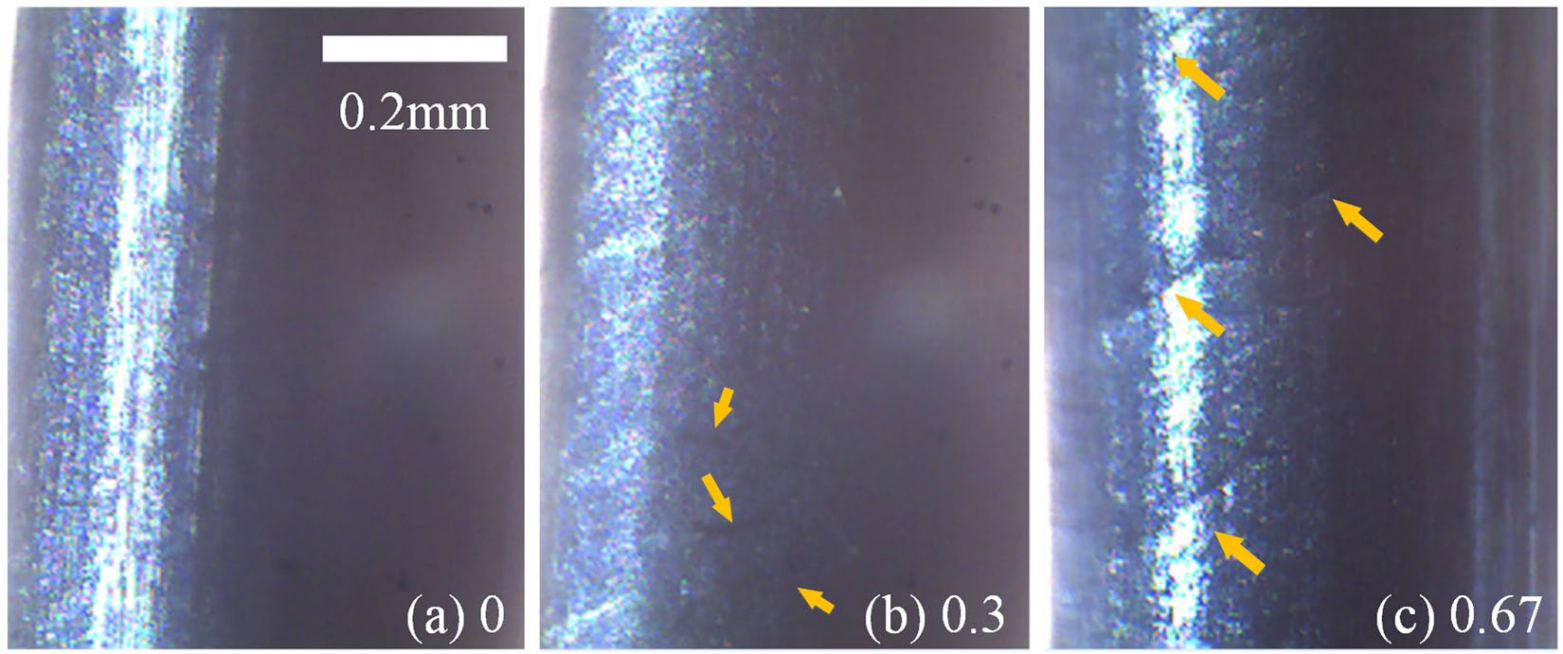
Microcracks on the surface of wires in different fatigue stages, which were observed by an optical microscope. (**a**) The wire was not bent. (**b**) The *N*/*N*_*m*_ ratio was 0.3. (**c**) The *N*/*N*_*m*_ ratio was 0.67.

The decrease of the photoacoustic eigen-frequency could be related to the mechanical changes due to low-cycle fatigue. The high degree of bending could cause dislocation rearrangement and microcracks in the wires. We have observed the microcracks on the surface of wires in different progresses of fatigue by using an optical microscope, as shown in Fig. 6. Figure 6(a–c) correspond to situations that the *N*/*N*_*m*_ ratio equals 0, 0.3 and 0.67, respectively. It is clear that there was not any crack on the wire before it was bent. But the amount of cracks increased and the lengths of cracks got longer after different bending degrees. These observations show that microcracks develop with the accumulation of fatigue in our fatigue experiment. In the low-cycle fatigue, plastic deformation happens and accompanies with the microstructural changes, especially the increase of microcracks and dislocation density^51^. The cracks and dislocation due to fatigue damage will decrease the elastic modulus^34,52^ and thus decrease the eigen-frequencies. Both the microcracks and dislocations could contribute to the frequency decrease. The reduction in the wire diameter induced by the fatigue test could be another factor that affects the resonance frequency. We measured the wire diameter as the fatigue accumulated during the test but did not find observable changes in diameter. Moreover, the theory predicts that the decrease in diameter will increase the resonance frequency. Thus, the decrease in the resonance frequency observed in our experiments was essentially caused by mechanical changes due to fatigue, but not diameter changes.

Based on the above observations and analyses, the basic mechanism can be summarized as follows: fatigue test causes the development of microcracks and dislocation in wires. Then the changes of microstructure reduce the elastic modulus, and thus decrease the eigen-frequencies. Inversely, the decrease of eigen-frequencies observed in photoacoustic signals could reflect the variation of the mechanical characteristics caused by low-cycle fatigue, thus enabling the monitoring of low-cycle fatigue. Therefore, based on the noncontact measurement of the photoacoustic eigen-spectrum and quantification of the eigen-frequency shifts, we could characterize the low-cycle fatigue damage of the samples.

## Discussion

In summary, we propose a method for noncontact and noninvasive low-cycle fatigue characterization of cylinders via photoacoustic eigen-spectrum analysis. We theoretically predicted the eigen-frequencies of cylindrical optical absorbers. Photoacoustic eigen-spectra extracted from experimental photoacoustic waves confirm the theoretical predictions. We then performed photoacoustic measurements on steel wires with various degrees of low-cycle fatigue. Photoacoustic eigen-spectrum analysis examines the decreases in eigen-frequency induced by low-cycle fatigue. By using an optical microscope, we observed that the microcracks on the surface of wires develop with the accumulation of fatigue. In the low-cycle fatigue, plastic deformation occurs and accompanies with the microstructural changes, especially the increase of microcracks and dislocation density. The cracks and dislocation will decrease the elastic modulus, and thus reduce eigen-frequencies. Inversely, the decrease of eigen-frequencies observed in photoacoustic signals reflects the low-cycle fatigue, which has changed the microstructural and mechanical properties of metallic wires. Therefore, these results show that this method could detect low-cycle fatigue damage without contact and thus noninvasively. In this study, only samples with simple regular shapes and homogeneous materials properties were examined because the photoacoustic eigen-spectra of such samples can be easily predicted for experimental verification. Inhomogeneous or irregularly shaped samples involve more complicated eigen-frequency calculations, but the proposed method should still be applicable.

Many studies have monitored the mechanical characteristics and fatigue of various materials^30–45^. Some methods^30–34^ rely on contacting vibration excitation or contacting signal detection. All optical RUS^35–37^ and laser ultrasonic methods^38–41^ are contactless techniques for the evaluation of mechanics and fatigue. However, these methods require direct detection of the surface vibration of samples by optical methods. This requirement limits the implementation of fatigue assessment of materials embedded in optically scattering media, such as intravascular stents in deep tissues. EMAR shows a certain sensitivity and ability for the online monitoring of fatigue without interrupting fatigue tests^42–45^. However, this method is typically suitable only for ferromagnetic metals. Compared with these methods, the proposed photoacoustic technique offers certain advantages and various suitable applications. First, our method can test fatigue over a relatively long depth within optically turbid materials, such as tissues. Pulsed lasers can penetrate biological tissues at a depth of several centimeters and excite photoacoustic waves. This method could be applicable to monitoring mechanical changes, e.g., fatigue, deep within turbid media. Additionally, the photoacoustic eigen-spectrum might enrich the imaging contrast of PAI in terms of mechanical characterization. Second, our method can be easily applied to small specimens, such as the wires with a submillimeter diameter used in our study. Third, the photoacoustic method can be widely applied to specimens made of various materials and is not limited to ferromagnetic metals. Our method is based on the photoacoustic effect due to laser absorption. This effect is broadly observed in various materials. Therefore, in addition to ferromagnetic specimens, other materials, such as bone^9^ and titanium implants^23^, are suitable for detection using our method.

The final goal of this study was the use of the proposed method for biomedical applications. Certain problems should be considered before real-world application. Biological tissues are similar to water in density and speed of sound. However, they are also different in other ways; for example, tissue has a higher viscidity than water. This higher viscidity means stronger acoustic absorption, which will accelerate the attenuation of photoacoustic signals, particularly the high-frequency components. Although absorption has no significant influence on the eigen-frequency, the attenuation of the signal will decrease the detection distance. Additionally, the method proposed in this study relies on resonance; that is, the wire should be an ideal resonator. Otherwise, the resonance could be weak, and the eigen-frequencies could be difficult to detect, as in, for example, a superelastic NiTi alloy. This is a limitation of the proposed method. In addition to the use of a stronger pulsed laser to enhance photoacoustic-spectrum excitation, another possible solution is to use a sinusoidal continuous laser as the optical excitation source. The resonant spectrum could be measured by sweeping the modulation frequency of the laser. This avenue is worthy of future research.

## Methods

### Calculation of photoacoustic eigen-frequency

The calculation of eigen-frequencies evolves into the solution of the equation det(**D**) = 0. The elements of the matrix **D** are

$$\begin{array}{rcl}{d}_{11} & = & ({\rho }_{f}/{\rho }_{c}){k}_{s}^{2}{a}^{2}{H}_{n}({k}_{f}a),\\ {d}_{12} & = & -2{k}_{d}a{J}_{n}\text{'}({k}_{d}a)+(2{n}^{2}-{k}_{s}^{2}{a}^{2}){J}_{n}({k}_{d}a),\\ {d}_{13} & = & 2n[{k}_{s}a{J}_{n}\text{'}({k}_{s}a)-{J}_{n}({k}_{s}a)],\\ {d}_{21} & = & -{k}_{f}a{H}_{n}({k}_{f}a),\\ {d}_{22} & = & {k}_{d}a{J}_{n}\text{'}({k}_{d}a),\\ {d}_{23} & = & n{J}_{n}({k}_{s}a),\\ {d}_{31} & = & 0\\ {d}_{32} & = & 2n[{J}_{n}({k}_{d}a)-{k}_{d}a{J}_{n}\text{'}({k}_{d}a)],\\ {d}_{33} & = & 2{k}_{s}a{J}_{n}\text{'}({k}_{s}a)+({k}_{s}^{2}{a}^{2}-2{n}^{2}){J}_{n}({k}_{s}a).\end{array}$$

*J*_*n*_ and *H*_*n*_ are the *n*-th Bessel and Hankel functions, respectively. The relations indicate that matrix **D** depends on *λ*, *μ*, *ρ*_*c*_, *a*, *f*, *ρ*_*f*_ and *c*_*f*_, where *a* is the radius, and *f* is the frequency, *ρ*_*f*_ and *c*_*f*_ are the density and sound speed of the exterior media, respectively. The wave numbers *k*_*f*_, *k*_*s*_ and *k*_*d*_ are expressed as 2π*f*/*c*_*f*_, 2π*f*/*c*_*s*_ and 2π*f*/*c*_*d*_. It is clear that the eigen-frequency depends on the mechanical properties of the cylindrical optical absorber. By defining a function *φ*(*f*) = det(**D**), the eigen-frequencies are the roots of the equation *φ*(*f*) = 0. Using Newton’s iteration formula *f*_*i*+1_ = *f*_*i*_ – *φ*(*f*_*i*_)/*φ′*(*f*_*i*_) and choosing the appropriate initial values, we can obtain the complex eigen-frequencies. The imaginary parts denote the attenuation and are of no significance; they are thus ignored in the analysis.

### Data availability

The datasets generated during and/or analysed during the current study are available from the corresponding author on reasonable request.
